# Supplementary material for: Direct cell interactions potentially regulate transcriptional programmes that control the responses of high grade serous ovarian cancer patients to therapy
Source: Sci Rep. 2025 Apr 25;15:14484. doi: 10.1038/s41598-025-98463-5 (PMC12032223; doi:10.1038/s41598-025-98463-5)

SUPPLEMENTARY FIGURE 1

a. Complete remission (CR) network

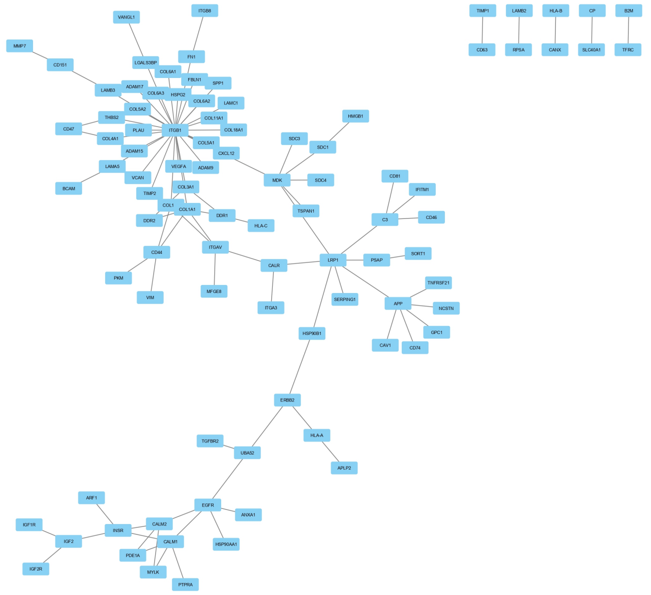

b. Partial remission (PR) network

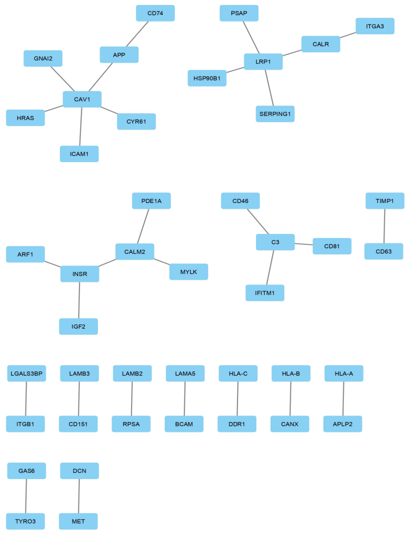

c. Progressive disease (PD) network

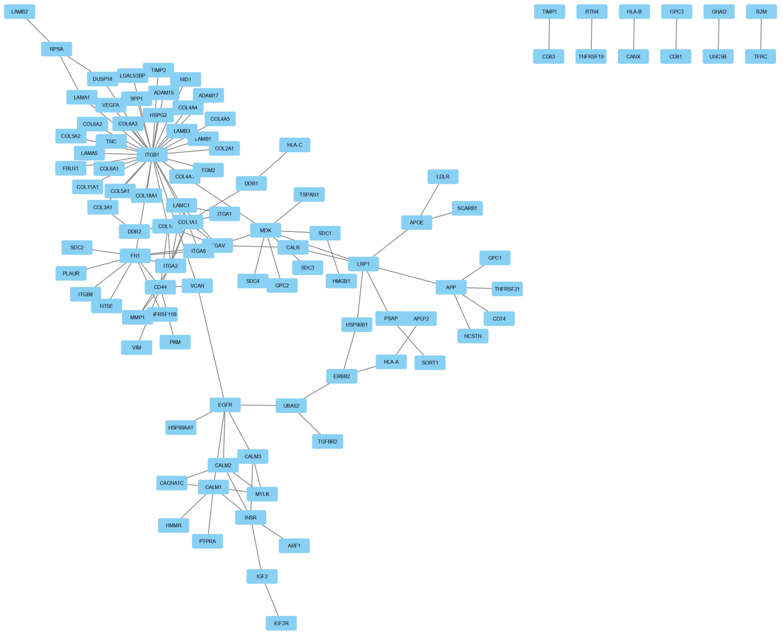

Supplement: Supplementary file 3 — Supplementary Information 3. [file 41598_2025_98463_MOESM3_ESM.pdf]
