# Supplementary figures and images for: Direct cell interactions potentially regulate transcriptional programmes that control the responses of high grade serous ovarian cancer patients to therapy
Source: Sci Rep. 2025 Apr 25;15:14484. doi: 10.1038/s41598-025-98463-5 (PMC12032223; doi:10.1038/s41598-025-98463-5)

SUPPLEMENTARY FIGURE 2

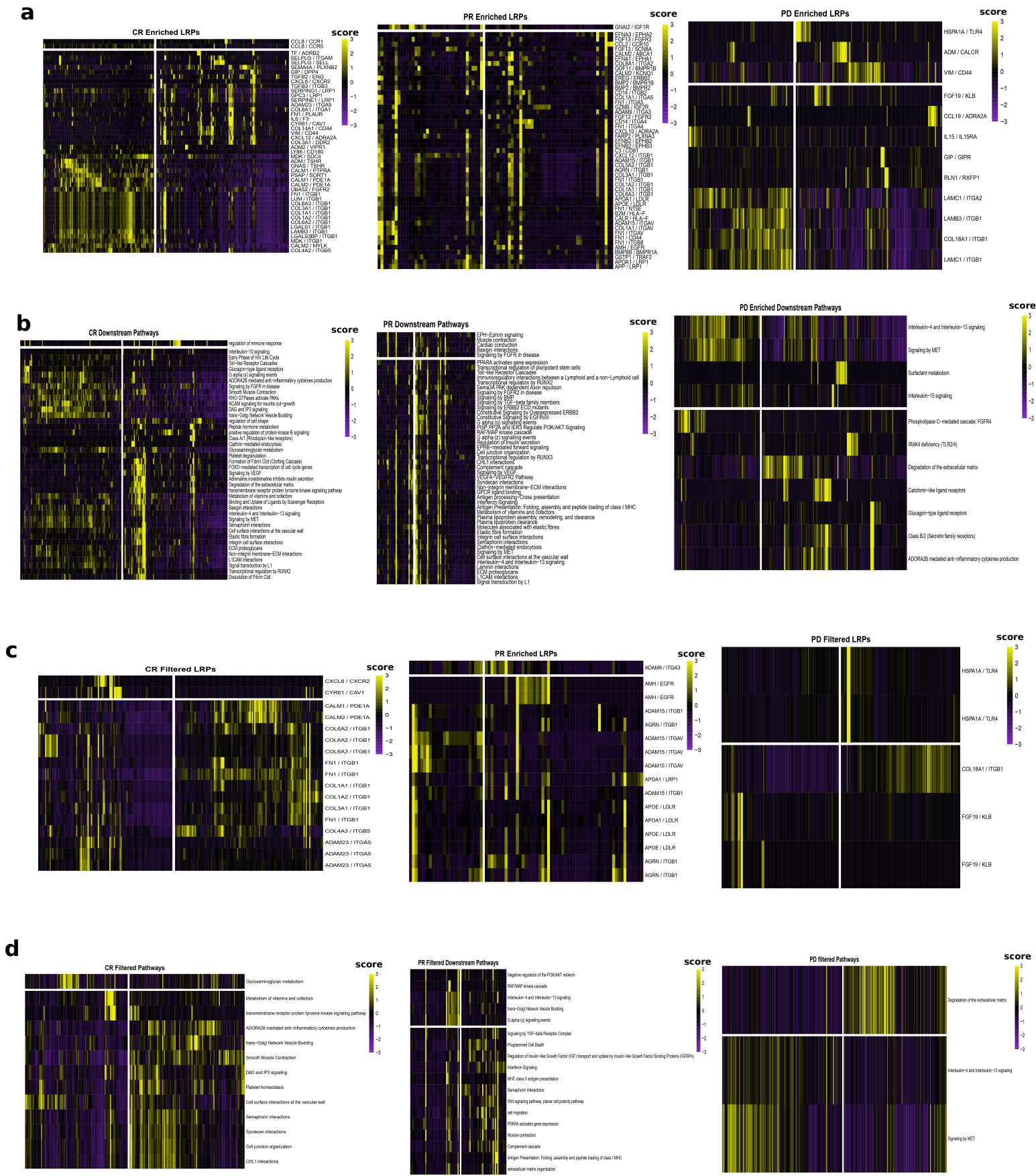

Supplement: Supplementary file 4 — Supplementary Information 4. [file 41598_2025_98463_MOESM4_ESM.pdf]

SUPPLEMENTARY FIGURE 3

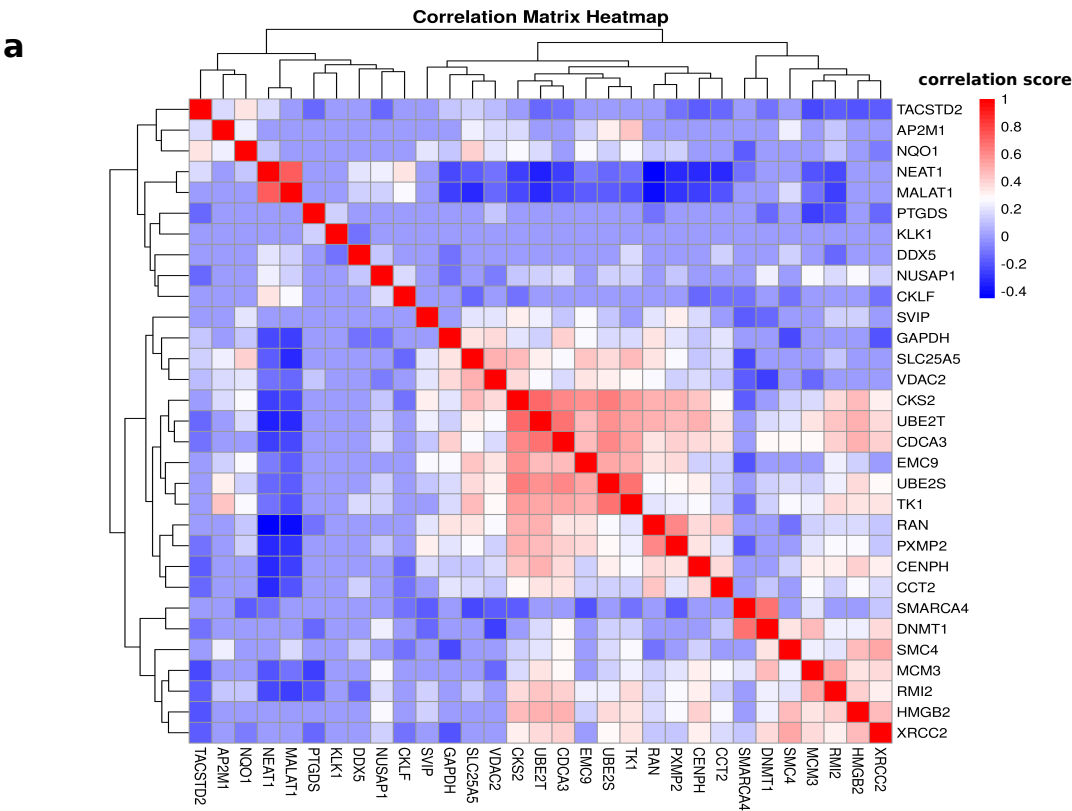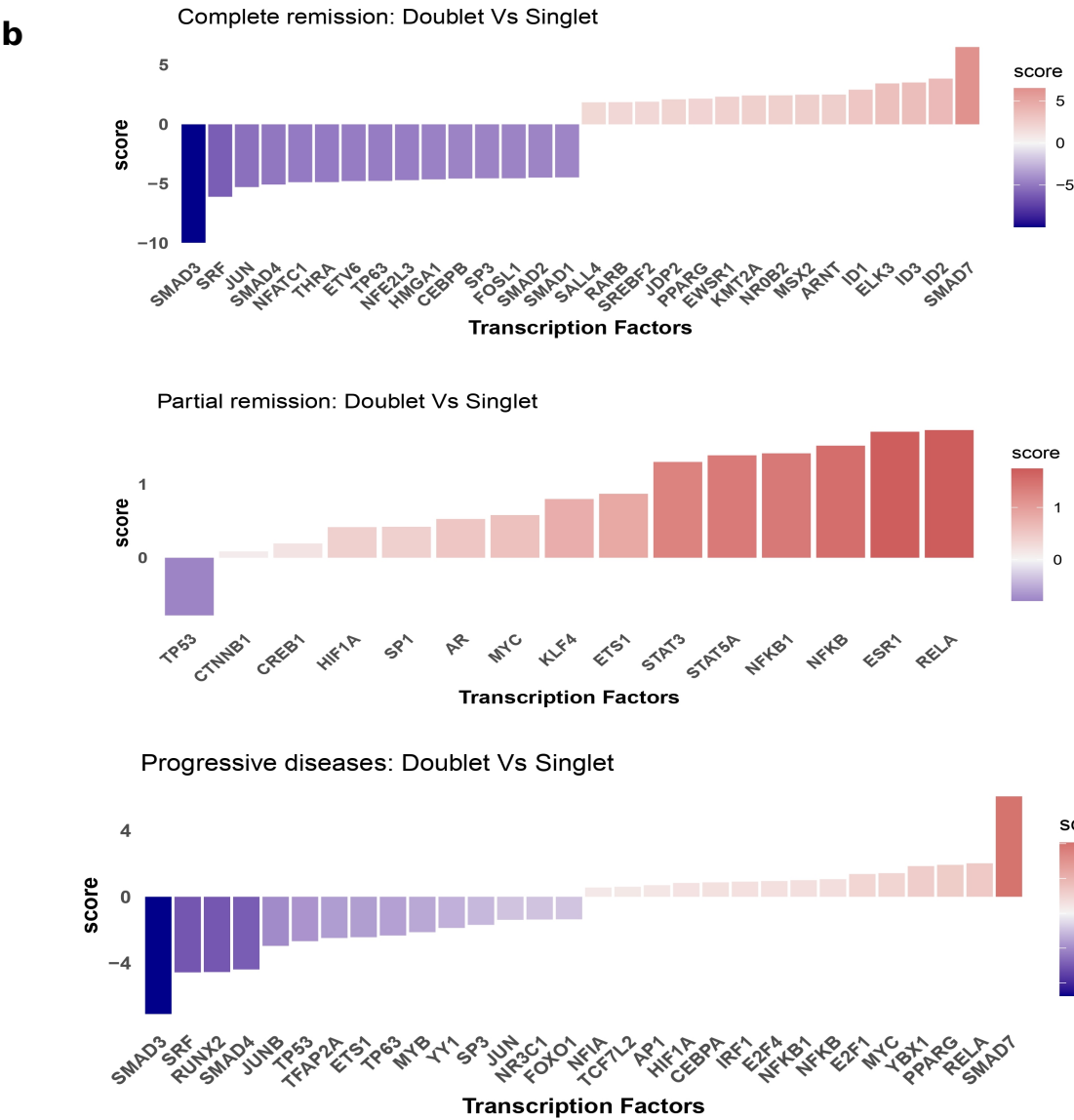

Supplement: Supplementary file 5 — Supplementary Information 5. [file 41598_2025_98463_MOESM5_ESM.pdf]
