# Supplementary material for: Direct cell interactions potentially regulate transcriptional programmes that control the responses of high grade serous ovarian cancer patients to therapy
Source: Sci Rep. 2025 Apr 25;15:14484. doi: 10.1038/s41598-025-98463-5 (PMC12032223; doi:10.1038/s41598-025-98463-5)
Supplement: Supplementary file 6 — Supplementary Information 6. [file 41598_2025_98463_MOESM6_ESM.docx]

**SUPPLEMENTARY FIGURE LEGENDS**

**Supplementary figure 1: Ligand receptor interaction network**

A manual scoring approach was utilised to infer ligand receptor co-expression in cancer-stromal doublets. Here, an average expression of each gene was calculated from the normalised expression data. A list of publicly available ligand-receptor pairs was obtained containing 708 unique ligands and 691 unique receptors (Ramilowski et al., 2015). An enrichment score for each ligand or receptor gene in a doublet cluster was calculated and filtered on certain parameters (see methods). The resulting ligand receptor pairs were visualised as a ligand-receptor interaction network for (a) complete remission, (b) partial remission, and (c) progressive diseases using Cytoscape v3.10.0.

**Supplementary figure 2: Inferred ligand-receptor pairs and corresponding downstream pathways**

a. Active ligand-receptor pairs (LRPs) inferred from cancer-stromal doublets for complete remission (left), partial remission (middle) and progressive disease (right) using BulkSignalR. For a ligand-receptor interaction pair to be considered active, the corresponding downstream signalling targets must have a significantly correlated expression level.

b. The downstream signalling pathways driven by the inferred LRPs (above) in the cancer-stromal doublets for complete remission (left), partial remission (middle) and progressive disease (right)

c. Filtered LRPs representing the overlap between the LRPs inferred above and those obtained using interaction scoring approach (see main text, section 3.3) for complete remission (left), partial remission (middle) and progressive disease (right).

d. The downstream signalling pathways driven by the filtered LRPs (above) in the cancer-stromal doublets for complete remission (left), partial remission (middle) and progressive disease (right).

**Supplementary figure 3: Gene Correlations and Transcription Factor Activity**

a. A heatmap of the correlation scores between genes that were significantly associated with clinical prognosis from multivariate Cox regression analysis of a cohort of 399 advanced stage ovarian cancer patients. The p values were adjusted using the false discovery ratio correction method. Genes with adjusted p values < 0.05 were considered significant. Light blue boxes represent insignificant correlations (zero).

b. Differential TF activities of doublets compared to singlets for complete remission (top), partial remission (middle), and progressive disease (bottom). For each clinical outcome, a differential expression analysis was performed between the cancer-stromal doublets against the singlet cancer cells and stromal cells to obtain the differentially expressed genes. TF activities were then inferred from significantly differentially expressed genes. Significant genes are those with p-values < 0.05.
